# Engineered exosomes: a promising drug delivery platform with therapeutic potential

**DOI:** 10.3389/fmolb.2025.1583992

**Published:** 2025-05-09

**Authors:** Genevieve Schwarz, Xuechen Ren, Wen Xie, Haitao Guo, Yong Jiang, Jinyu Zhang

**Affiliations:** ^1^ Department of Cell and Cancer Biology, The University of Toledo College of Medicine and Life Sciences, Toledo, OH, United States; ^2^ Center for Pharmacogenetics and Department of Pharmaceutical Sciences, University of Pittsburgh, Pittsburgh, PA, United States; ^3^ Department of Pharmacology and Chemical Biology, University of Pittsburgh, Pittsburgh, PA, United States; ^4^ Department of Microbiology and Molecular Genetics, Cancer Virology Program, UPMC Hillman Cancer Center, University of Pittsburgh, Pittsburgh, PA, United States

**Keywords:** Exosomes, cardiovascular diseases, liver fibrosis, immune diseases, and nervous disorders

## Abstract

Exosomes, small membranous vesicles naturally secreted by living cells, have garnered attention for their role in intercellular communication and therapeutic potential. Their low immunogenicity, high biocompatibility, and efficient biological barrier penetration make them promising drug delivery vehicles. This review spans research developments from 2010 to 2025, covering the engineering of exosomes to optimize cargo loading and targeting specificity. We discuss their applications in treating cardiovascular diseases, liver fibrosis, immune diseases, and neurological diseases, alongside ongoing clinical trials and industry progress. Future challenges include scalability, standardization, and minimizing off-target effects. We propose strategies to address these hurdles, such as bioengineering techniques and improved isolation methods. By synthesizing current knowledge and outlining future directions, this review aims to guide researchers toward harnessing exosomes for disease treatment.

## 1 Introduction

**FIGURE 1:**
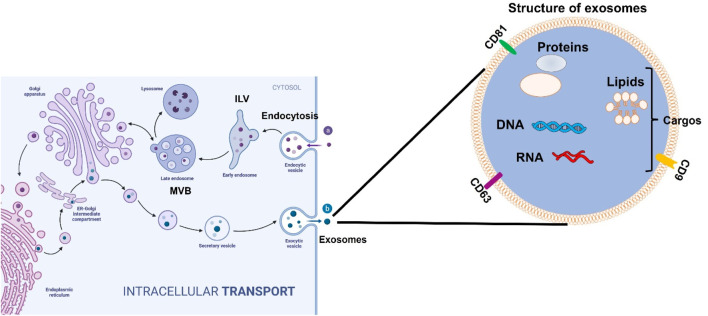
The structure and formatting process of exosomes secreted from cells. In the beginning, exosomes form with the inward budding of the cytoplasmic membrane. Exosomes contain various molecules, including lipids, proteins, DNA, and RNA. These vesicles then turn into intraluminal vesicles (ILVs) as the early stage of exosomes and later develop into a late stage called multivesicular bodies (MVBs). Finally, the MVBs can be degraded inside the cells by lysosomes or autophagosomes. MVBs can directly fuse the plasma membrane where ILVs are released to the intercellular space between cells as exosomes to regulate the downstream signaling pathways.

Exosomes are small, endosome-derived membrane microvesicles released by all types of prokaryotic and eukaryotic cells. They play a key role in regulating cellular functions and facilitating intercellular communication across various disease types (Deatherage and Cookson, 2012; Doyle and Wang, 2019). Their intrinsic properties support communication between cells and tissues and enable them to regulate complex intracellular pathways in many pathological conditions. The important role of exosomes in intercellular communication is closely tied to their classification as extracellular vesicles (EVs). Typically ranging in size from 30 to 150 nm in diameter, exosomes originate from the inward budding of the limiting membrane of early endosomes, eventually forming multivesicular bodies (MVBs) (Figure 1). Exosomes can migrate from cell to cell through three mechanisms (Han et al., 2016). First, the ligands on the exosomal membrane directly bind to the target cells’ membrane and then regulate the intracellular signaling pathways. Second, the exosome membrane can directly fuse with the recipient cells’ membrane and release its contents into the recipient cell. Third, recipient cells can directly phagocytose exosomes and incorporate them into their ingredients. The size range and characteristics of exosomes enable them to deliver a variety of bioactive molecules, including lipids, proteins, metabolites, and nucleic acids, which influence key signaling processes in target cells. Exosome-mediated cargo delivery plays a critical role not only in disease diagnosis—serving as diagnostic biomarkers for early detection, disease progression, and prognosis in conditions such as cancer, neurodegenerative diseases, and cardiovascular disorders—but also in the targeted delivery of pharmaceutical agents and bioactive compounds to specific organs and cells (Chen et al., 2021b).

## 2 The biogenesis and function of exosomes

The first recognition of exosomes occurred in 1983 when exosomes were discovered to be fundamental in cell-to-cell communication, and physiological, and pathological homeostasis in tissues (Edgar, 2016). As shown in Figure 1, the formation of exosomes starts from the inward budding of the plasma membrane to form early endosomes, and then these early endosomes mature and develop into late endosomes in the cytosol. These late endosomal membranes invaginate and form intraluminal vesicles (ILVs), and in this process, some contents, such as lipids, proteins, DNA, and different RNAs including messenger RNA (mRNA), short single-stranded microRNAs, long non-coding RNAs, and novel circular RNAs (circRNA) from the host cells will be engulfed into future exosomes. At this point, the ILVs incorporate the endosomal sorting complex required for transport (ESCRT) machinery, tetraspanins, or lipid-dependent processes that are needed for the formation of MVBs (multivesicular bodies) following the formation of ILVs. MVBs as late exosomes are then either consumed by lysosomes or fused with the plasma membrane via the actin cytoskeletal and microtubule network. The vesicles undergo exocytosis where the ILVs get secreted as exosomes (Chen et al., 2021b).

**FIGURE 2:**
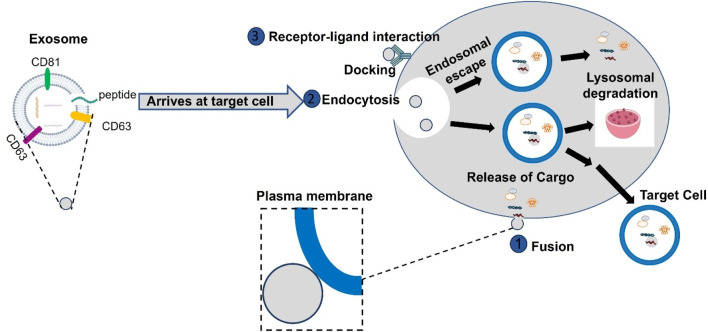
Exosomes interact with target cells through multiple pathways, primarily in three ways. They can be internalized by target cells via endocytosis, after which they fuse with the endosome membrane to release their contents into the cytoplasm. Alternatively, exosomes may directly fuse with the target cell membrane. Additionally, exosomes can directly enter target cells through receptor-ligand interactions, where bioactive ligands on the exosome surface bind to specific receptors on the target cells, facilitating the delivery of their contents.

Generally, the interaction between exosomes and the target cells occurs, through three different mechanisms. At times, exosomes interact with the target cell, through micropinocytosis, incorporating into the cell through the invagination of the target cell plasma membrane as shown in Figure 2. In other cases, the interaction between exosomes and target cells occurs through ligand-receptor binding as shown in Figure 2. A cascade response is stimulated through the ligand-receptor binding that occurs on the surface of the target cell releasing the cargos delivered by exosomes. Alternatively, exosomes may directly fuse with the target cell membrane. Regardless of which mechanism exosomes will employ, they present an extremely favorable strategy for the delivery of molecules (Chen et al., 2021b). Based on the above biogenesis and functions of exosomes, in this review, we endeavor to update the novel and latest information on exosome biogenesis, molecular properties, and functional activities in various diseases reported so far. In addition, we discussed the dual role of exosomes as clinical biomarkers and the therapeutic potential of engineered exosomes as vehicles for specifically targeted therapy in cardiovascular diseases, liver fibrosis, immune diseases, and nervous disorders.

**FIGURE 3:**
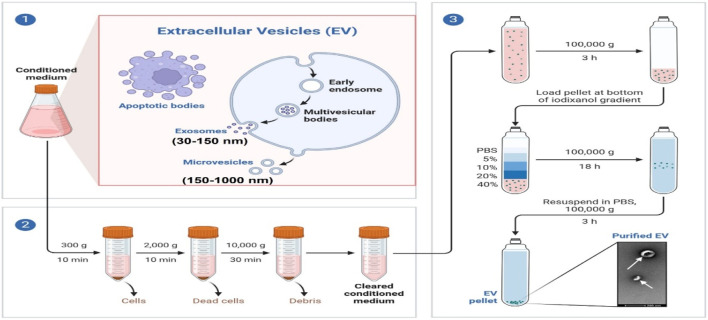
Schematic representation of exosome purification by differential ultracentrifugation. Culturing different types of cells and collecting culture medium, including apoptotic bodies, exosomes, and microvesicles. Exosomes are isolated by differential ultracentrifugation from low to high. The purified exosomes are characterized by size and morphology through TEM.

Based on the above biological, physical, and chemical characterizations, several approaches have been applied to isolate and purify exosomes. These approaches include ultracentrifugation, ultrafiltration, column chromatography, and microplate-based magneto-immunocapture using commercial kits that have been established in many groups (Li et al., 2017; Coumans et al., 2017). However, the most prevalent method to isolate exosomes from culturing cell supernatant is differential ultracentrifugation. Although there are different identification criteria for exosome isolation in different systems, differential ultracentrifugation is still widely used because of the effective and simple handling method (Jeppesen et al., 2014). The procedure of exosome isolation from cells’ culture medium using the ultracentrifugation method is shown in Figure 3. Currently, the size, number, and characterization of isolated exosomes can be validated by nanoparticle tracking analysis (NTA), BCA method for measuring the concentration of exosomes and transmission electron microscopy (TEM) for the size and morphology of exosomes; additionally, Western blotting can also be used to detect exosomes by using different proteins enriched in exosomes, including ESCRT-related proteins (Tsg101 and Alix), surface proteins (CD81, CD63, and CD9) that have been identified as specific markers of exosomes, cytoplasmic proteins, and heat shock proteins (Hsp90 and Hsp70) (Ye et al., 2020).

## 3 Exosomes are more potential compared with other delivery systems

Exosomes, ectosomes, and enveloped viral particles (EVPs) are all extracellular vesicles with the capacity to transport biomolecules and facilitate intercellular communication. Despite their shared function, they differ significantly in their sizes, origins, cargo, and potential for therapeutic and diagnostic applications (Ahmadi et al., 2023). Exosomes are small vesicles (30–150 nm in diameter) derived from the inward budding of multivesicular bodies (MVBs) within cells, which then fuse with the plasma membrane to release their contents. Their cargo is diverse, including lipids, proteins, RNA, and metabolites, which can modulate the behavior of recipient cells, influencing processes such as immune response, tissue repair, and disease progression. Therapeutic Potential: Due to their natural biocompatibility and ability to cross biological barriers with minimal immune response, exosomes are being extensively explored as drug delivery vehicles (Li et al., 2025). Their ability to target specific cells or tissues with high precision makes them promising candidates for delivering pharmaceutical agents, including small molecules, RNA, and proteins, directly to diseased cells (Ferreira et al., 2022).

Ectosomes (also known as microvesicles) are larger vesicles (100–1000 nm in diameter) released by direct outward budding of the plasma membrane. Unlike exosomes, which are formed within intracellular compartments, ectosomes are shed from the cell’s surface. These vesicles can carry a range of biomolecules, including proteins, lipids, and RNA, and play roles in processes such as inflammation, coagulation, and cancer metastasis (Meldolesi, 2018; Wei et al., 2021). Therapeutic Potential: Ectosomes’ larger size and abundance of surface proteins make them valuable for targeted delivery, particularly for molecules requiring membrane fusion to enter cells. While they offer advantages for certain applications, such as immune modulation, their larger size can limit tissue penetration compared to exosomes, which may restrict their use in certain therapeutic contexts (Han et al., 2024b).

Enveloped viral particles (100–200 nm) are viral structures, composed of a lipid bilayer derived from the host cell membrane, enclosing the viral genome. These particles are naturally adept at delivering genetic material into host cells through membrane fusion, making them ideal candidates for gene therapy (He et al., 2022; Janns and Mikkelsen, 2024). Therapeutic Potential: EVPs can be engineered to deliver not only genetic material but also other therapeutic cargo, such as proteins or small molecules. However, the viral nature of these particles introduces safety concerns, such as immunogenicity and the potential for unintended viral replication. While EVPs are efficient at gene delivery, their use as cell delivery vehicles requires careful design to mitigate risks associated with their viral components (Huang et al., 2024). Taken together, exosomes, ectosomes, and enveloped viral particles each have distinct advantages and limitations as cell delivery vehicles. Exosomes stand out for their biocompatibility and precision in drug delivery, making them ideal for therapeutic and diagnostic applications as shown in Table1. Ectosomes, with their larger size and surface proteins, offer opportunities for targeted delivery in specific contexts. Meanwhile, enveloped viral particles, though effective for gene delivery, carry more substantial safety concerns due to their viral nature. Ongoing research into the engineering and modification of these vesicles holds great promise for advancing targeted therapies and drug delivery systems.

**TABLE 1:** Exosome application in various diseases.

Disease category	Exosome applications and mechanisms	Key references
Cardiovascular Diseases	Promoting cardiac repair and regeneration: Exosomes from mesenchymal stem cells (MSCs) deliver microRNAs (e.g., miR-21, miR-126, miR-146a) that reduce cardiomyocyte apoptosis and enhance proliferation. Enhancing angiogenesis: Exosomal VEGF, HIF-1α, and miR-210 stimulate endothelial cell proliferation and vascularization in ischemic heart diseases. Reducing myocardial ischemia-reperfusion injury: Exosomes from cardiac progenitor cells carry protective molecules like HSP70 and HSP27, which reduce oxidative stress and inflammation. Modulating immune responses: Exosomes derived from regulatory T cells (Tregs) suppress inflammatory cytokines (IL-6, TNF-α) in atherosclerosis and myocarditis	Lai et al. (2010), Bhaskara et al. (2023), Wang et al. (2017), Bai et al. (2018), Takeuchi et al. (2015), Zhuang et al. (2022), Xu et al. (2022)
Liver Fibrosis	Inhibiting hepatic stellate cell (HSC) activation: Exosomes from hepatocytes and MSCs deliver miR-19b and miR-29a, which suppress TGF-β1/SMAD signaling, reducing fibrotic activity. Reducing collagen deposition and fibrosis progression: Exosomal TIMP-1 and TIMP-2 inhibit matrix metalloproteinases, preventing excessive ECM accumulation. Delivering anti-fibrotic agents: Engineered exosomes loaded with siRNA targeting TGF-β1 or CTGF have been shown to reduce fibrosis in preclinical models. Modulating immune responses: Exosomal IL-10 and TGF-β reduce Kupffer cell-induced inflammation, mitigating liver injury	Devhare et al. (2017), Alshanwani et al. (2022), Shimoda and Khokha (2017), Borges et al. (2013), Wang et al. (2024), Wang et al. (2023), Feng et al. (2025)
Immune Diseases	Regulating immune cell functions: Dendritic cell-derived exosomes (DEX) carry MHC-II and co-stimulatory molecules, enhancing antigen presentation. Reducing autoimmune responses: Exosomes from tolerogenic dendritic cells suppress autoreactive T cells in diseases like rheumatoid arthritis and multiple sclerosis. Enhancing anti-inflammatory effects: Exosomes containing miR-150 and miR-21 inhibit NF-κB activation, reducing pro-inflammatory cytokine release. Acting as biomarkers: Circulating exosomal PD-L1 levels correlate with immune checkpoint activity in cancer and autoimmune diseases	Pitt et al. (2016), Xia et al. (2022), Zheng et al. (2024), Ricklefs et al. (2018), Feng et al. (2025)
Neurological Disorders	Facilitating neuronal repair: Neural stem cell-derived exosomes containing BDNF, NGF, and miR-132 enhance neuronal survival in ischemic stroke models. Delivering neurotrophic factors: MSC-derived exosomes transport cargo like GDNF, improving dopaminergic neuron survival in Parkinson’s disease. Reducing neuroinflammation: Exosomes carrying miR-124 suppress microglial activation, reducing neuroinflammatory damage in Alzheimer’s disease. Promoting remyelination: Oligodendrocyte-derived exosomes deliver MBP and PLP, supporting remyelination in multiple sclerosis models	Zhu et al. (2023), Vilaca-Faria et al. (2019), Ge et al. (2020), Zheng et al. (2024), Santos et al. (2024)

## 4 Role of exosomes in atherosclerosis

Cardiovascular diseases (CVDs), including atherosclerosis, stroke, and ischemic heart disease, are the leading cause of mortality worldwide, accounting for nearly one-third of deaths among older adults (Deatherage and Cookson, 2012). Current treatments often lack specificity due to the diverse genetic and molecular nature of CVDs, as well as the side effects associated with traditional gene therapy (Gould and Favorov, 2003). Therefore, there is a growing need for targeted therapeutic strategies, such as using engineered exosomes as delivery vehicles (Gould and Favorov, 2003; Spigel et al., 2021; Yuan and Huang, 2021; Chen et al., 2021a). Exosomes have shown remarkable potential in selectively interacting with target cells and efficiently delivering molecular cargo, thereby influencing cellular signaling and function (Reiss et al., 2023; Fu and Wu, 2021; Guo et al., 2020; Barile et al., 2017; Sahoo and Losordo, 2014). Given these capabilities, interest in exosome research for CVDs has increased significantly (Zhou et al., 2020a). In particular, exosome-derived microRNAs (miRNAs) have been identified as promising biomarkers for diagnosing heart disease and predicting myocardial injury (MI), heart failure, stroke, and endothelial dysfunction (Ciaccio and Tuttolomondo, 2023; Guan et al., 2022; Zheng et al., 2020). These advancements position exosomes as valuable tools for early disease detection, monitoring, and targeted drug delivery. However, their precise role in CVD pathophysiology remains unclear. Some studies suggest a direct correlation between exosome phenotyping and disease severity in CVD patients (Ma et al., 2021; Zamani et al., 2019; Zara et al., 2020). Further research is essential to elucidate the underlying mechanisms and develop exosome-based therapeutic strategies.

**FIGURE 4:**
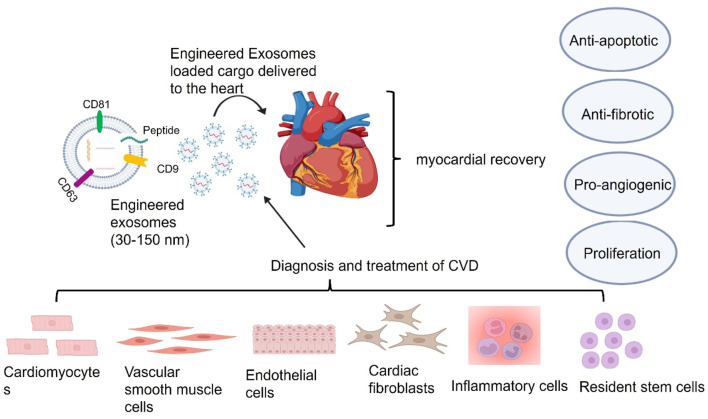
Exosomes derived from cardiac cells influence heart function through apoptosis regulation, fibrosis modulation, and angiogenesis support. Exosomes derived from different cell types in the heart, including cardiomyocytes, endothelial cells, vascular smooth muscle cells, cardiac fibroblasts, immune cells, and resident stem cells, can influence heart functions *via* the delivery of their cargos including nucleic acid, lipid, and protein. These cargos delivered by exosomes can reach all kinds of cells including cardiomyocytes, vascular smooth muscle cells, endothelial cells, cardiac fibroblasts and inflammatory cells and resident stem cells, in the heart *via* the bloodstream or directly through intracellular communication to employ their therapeutic benefits on apoptosis, fibrosis, and angiogenesis for myocardial recovery.

Atherosclerosis, a primary contributor to CVD, involves complex intercellular communication, in which exosomes play a key role (Vasan et al., 2021; Heo and Kang, 2022). Emerging evidence suggests that exosomes mediate different stages of atherosclerosis development and progression (Heo and Kang, 2022). They influence key cardiac cell types, including vascular smooth muscle cells (VSMCs), endothelial cells (ECs), and macrophages, to regulate atherosclerotic processes. For instance, endothelial cell-derived exosomes can activate CD137 inflammatory signaling, promoting VSMC proliferation and migration, leading to intimal hyperplasia and plaque formation after arterial injury (Li et al., 2020a). Additionally, exosomes from cardiac stromal cells in heart failure patients exhibit diminished regenerative capacity in MI models. However, loading these exosomes with specific DNA and RNA molecules, such as miR-21-5p, can restore regenerative function (Qiao et al., 2019), as illustrated in Figure 4. A notable study engineered interleukin-10 (IL-10) mRNA to include an internal ribosome entry site (IRES) responsive to microRNA-155 (miR-155), which is upregulated in inflamed atherosclerotic plaques. This engineered mRNA was encapsulated within exosomes and administered to ApoE^−/−^ mice, a model for atherosclerosis. The exosomes effectively delivered the IL-10 mRNA to macrophages within the plaques, where the presence of miR-155 triggered the translation of IL-10 protein. This targeted approach led to a significant reduction in atherosclerotic lesions with minimal off-target effects, demonstrating the promise of exosome-mediated, inflammation-responsive therapies for atherosclerosis (Bu et al., 2021). Additionally, exosomes have been explored as carriers for atheroprotective microRNAs (miRNAs) to repair damaged tissues caused by atherosclerosis (Lin et al., 2021). The circulating exosomes in patients with CADs promote atherosclerosis by facilitating the adhesion of pro-inflammatory cells and stimulating inflammatory responses, suggesting that targeting exosome-mediated pathways could be a viable therapeutic strategy (Han et al., 2024a). Stem cell-derived exosomes have been highlighted the role in managing atherosclerosis which can modulate key pathophysiological pathways, including inflammation, angiogenesis, and cellular senescence, which are critical in the development of atherosclerosis (Tariq et al., 2025). The engineered mesenchymal stem or stromal cell-derived exosomes designed for targeted drug delivery in cardiovascular diseases, including atherosclerosis. These engineered exosomes can be modified to carry specific therapeutic agents, enhancing their potential as targeted delivery systems (Bu et al., 2021). Therefore, these advancements underscore the therapeutic potential of exosome-based delivery systems in atherosclerosis, offering targeted and efficient treatment modalities as shown in Table1.

## 5 Immune diseases and exosomes

**FIGURE 5:**
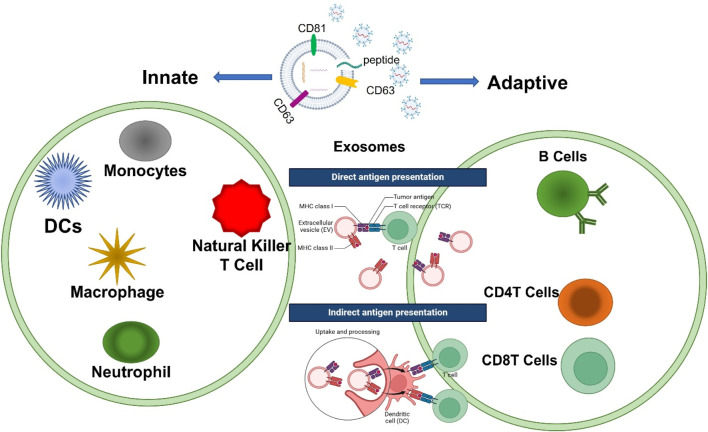
Exosomes contribute to the basic processes of innate and adaptive immunity. The immunoregulatory functions of exosomes mainly include affecting antigen presentation directly or indirectly to regulate the development of B cells and the activation of T cells including CD4T and CD8T cells, immunosuppression, the inflammatory response, and intercellular communication in different immune cells.

Exosomes participate in many cellular processes including immune responses (Barros et al., 2018), signal transductions (Bahrami et al., 2021), and antigen presentations in different immune cells (Lindenbergh et al., 2020). Over the past 5 years, exosomes have been proven to be more than just mRNA carriers and are part of a dynamic interchange between cells during communication (Maas et al., 2017). Exosomes can modulate the communication in immune cells across the innate and adaptive immune systems as shown in Figure 5. In the adaptive immune system, exosomes are the leading component that provides NOX2 membrane clusters to CD4^+^ T cells to restrain the activation and expansion of CD4^+^ T cells (Akiyama et al., 2020). In innate immunity, exosomes are responsible for the release of IFNI and the activation of dendritic cells (DCs) (Zhou et al., 2020b). Subsequently, scientists began to conceptualize innovations in applying exosomes to aid the immune system. CRISPR/Cas9 was packaged into exosomes in a successful effort to suppress the angiogenesis of tumors by reducing the concentration of miR-494 in a study on lung cancer (Shao et al., 2021). Studies focused on sepsis have shown that exosomes derived from B cells can lower the release of macrophage-derived pro-inflammatory factors by acting as carriers of synthetic miRNA inhibitors (Qiu et al., 2021). A recent study has identified that exosomes containing STING (Stimulator of Interferon Genes) aid the immune response by reducing tumor growth and are associated with the proliferation of CD8^+^ T cells (McAndrews et al., 2021). As shown in Figure 5, immune cells involved in innate immunity, including macrophages, DCs, natural killer (NK cells), and granulocytes, can recognize antigens through a class of pattern recognition receptors (PRRs) to induce immune responses. Furthermore, exosomes can have a function in the polarization of macrophages (Sun et al., 2018), the regulation and processing maturation of DCs (Zhou et al., 2014), and the cytotoxic function of NK cells (Li et al., 2018b; Borrelli et al., 2018). T and B cells involved in processes of adaptive immunity are activated, proliferate, and differentiate into effector cells after receiving antigen stimulation and cause a series of biological effects in cells. Reversely, engineered exosomes participate in adaptive immune responses by disseminating antigens or major histocompatibility complex (MHC) peptide complexes to stimulate the development of dendritic cells presenting them or to directly communicate with memory T cells by uptaking (Zou et al., 2021). As research continues to uncover their full potential, exosomes are poised to become powerful tools for both diagnostic and therapeutic applications in immune-related diseases as shown in Table1. Understanding their mechanisms and optimizing their use will be key to harnessing their benefits in clinical settings.

## 6 Exosomes in liver fibrosis

Liver fibrosis (LF) is a global health concern characterized by excessive scarring in the liver due to hepatocyte damage caused by various factors, including hepatic viral infections, alcohol consumption, and metabolic disorders. Hepatocyte damage triggers the transition of quiescent hepatic stellate cells (HSCs) into activated HSCs, leading to extracellular matrix (ECM) deposition, which is central to the initiation and progression of LF. In addition to HSC activation, hepatic macrophages and hepatocytes secrete cytokines that contribute to fibrosis progression (Roehlen et al., 2020; Aydin and Akcali, 2018).

Under normal physiological conditions, HSCs reside in the liver in a quiescent state, playing a crucial role in vitamin A homeostasis, lipid storage, and the synthesis of matrix metalloproteinases (MMPs). However, during chronic liver injury, HSCs undergo activation and transdifferentiate into proliferative myofibroblast-like cells characterized by increased expression of α-smooth muscle actin (αSMA) and collagen. Myofibroblasts are the primary source of ECM production, making HSC activation a key regulatory factor in LF progression (Luo et al., 2021b). Various stressors, including alcohol, lipids, and hepatitis B/C virus infections, can induce fatty liver disease, with exosomes derived from hepatocytes playing a pivotal role in activating quiescent HSCs into myofibroblasts (Safran et al., 2022). Notably, lipotoxic exosomal miR-1297 from primary hepatocytes has been shown to promote HSC activation and proliferation via the PTEN/PI3K/AKT signaling pathway, accelerating metabolic-associated LF (Luo et al., 2021c; Luo et al., 2021d).

Viral infections, particularly hepatitis B virus (HBV) and hepatitis C virus (HCV), are significant contributors to LF. Exosomal miRNAs have been identified as potential biomarkers in this process (Liu et al., 2023b). During HBV-induced liver fibrosis in mice, exosomes from HBV-infected hepatocytes contribute to HSC activation. Exosomal miR-222 enhances HSC activation by inhibiting transferrin receptor (TFRC)-induced HSC ferroptosis, highlighting the critical role of the miR-222/TFRC axis in LF progression (Zhang et al., 2023b). Similarly, exosomes from HCV-infected hepatocytes contain high levels of replication-associated miRNAs that drive fibrosis. Exosomal miR-19a, delivered from HCV-infected hepatocytes to HSCs, activates HSCs through the SOCS-STAT3 axis, while exosomal miR-192 promotes HSC activation in HCV-induced LF (Qiu et al., 2021). Additionally, exosomal miR-192 secreted from HCV-replicating hepatocytes to HSCs has been identified to promote HSC activation in HCV-induced LF (McAndrews et al., 2021). Therefore, HSC activation is essential for the processing of virus-induced liver fibrosis. Given the central role of HSC activation in viral-induced fibrosis, targeting and interrupting this process presents a promising therapeutic strategy.

**FIGURE 6:**
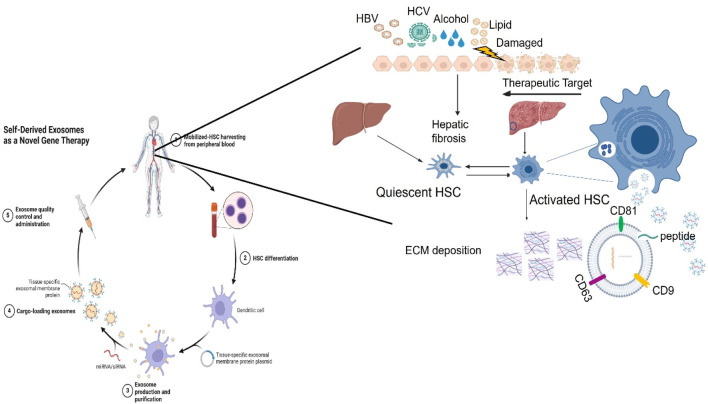
Exosomes contribute to liver fibrosis by targeting HSCs. Hepatocytes can be damaged due to many factors, including alcohol, lipids, and virus infection, specifically hepatitis virus (HBV/HCV). A variety of different pathways by different cells’ functions mediate chronic inflammation that exacerbates liver fibrosis. Immune cells including Kupffer cells, macrophages, and HSCs, secrete a lot of proinflammatory cytokines to promote the infiltration of inflammatory cells, and aggravate liver inflammation. As a result of liver injury, exosomes secreted by activated hepatocytes contain macromolecules that drive HSCs from quiescent HSCs to activated HSCs. Meanwhile, HSCactivation and proliferation directly drive extracellular matrix (ECM) deposition increases, resulting in liver fibrosis and liver dysfunction.

Exosomes derived from mesenchymal stem cells (MSCs) and other cultured cells have demonstrated anti-fibrotic effects. Serum-derived exosomes are promising biomarkers for LF diagnosis and have therapeutic potential in liver disease treatment. Exosomes are applied to therapeutic potential in treating liver disease (Liu et al., 2023a) as shown in Figure 6. Exosomes facilitate cell-to-cell communication by delivering various molecular contents that can either promote or inhibit HSC activation. Studies indicate that most liver-resident cells, whether normal or injured, secrete exosomes, influencing fibrosis progression (Thery et al., 2002). The quantity, contents, and biological characterization of released exosomes from different conditions can increase or inhibit the physiological or pathological progression of HSCs in liver fibrosis. However, hepatocytes were found to release a small number of exosomes to regulate liver repair and regeneration in the injury of the liver (Nojima et al., 2016). Meanwhile, stressed hepatocytes in an injury of the liver were found to induce exosome release and the expression of some cellular mRNAs, which modulates the transcriptional process of liver fibrosis (Chen et al., 2018). Exosomes from non-parenchymal cells, including endothelial cells, Kupffer cells, or HSCs, may be involved in liver regeneration and function during liver fibrosis processing. miR-122-containing exosomes can modulate the activation of hepatic stellate cells and contribute to liver regeneration (Chang et al., 2021). In addition, exosomes from liver tumor cells play an important role in tumor growth, vessel angiogenesis, tumor cell proliferation, and metastasis of neoplasm in liver tumors (Sung et al., 2018). Therefore, exosome-regulated immune responses can function as yin and yang in liver diseases (Kalluri and LeBleu, 2020).

Accumulating evidence has widely demonstrated that exosomes play a role in liver disease not only as the biomarker but also as the potential treatment tool (Fonsato et al., 2012; Liang et al., 2018). Due to their low immunogenicity and biocompatibility, exosomes can more efficiently and specifically deliver biomolecules to the target cells without degradation and loss of biological information for these biomolecules. Li et al. developed engineered exosomes to target specific RNAs for lysosomal degradation as a potential treatment for liver fibrosis (Li et al., 2020b). Notably, miR-155 is known for its pro-inflammatory effects that contribute to liver fibrosis (Salimi et al., 2018). Specifically, they created a fusion protein by attaching the RNA-binding protein HuR to the C-terminus of Lamp2b, a membrane protein present in both exosomes and lysosomes. This HuR-Lamp2b fusion protein was successfully incorporated into exosomes. The HuR-Lamp2b fusion protein targets miR-155, leading to its degradation and thereby exerting a protective effect against fibrosis (Bala et al., 2016). Therefore, developing fusion proteins to direct specific RNA degradation presents a promising therapeutic strategy for liver fibrosis.

CRISPR/Cas9 gene editing has been widely used in gene therapy for various diseases (Wong et al., 2022; Fujii et al., 2019). Recent studies have utilized exosomes to deliver functional proteins via the CRISPR/Cas9 system to treat liver fibrosis. For instance, hepatocyte nuclear factor 4α (HNF4α) regulates hepatocyte differentiation by controlling numerous hepatic genes (Colletti et al., 2009). In 2021, Luo’s team reported that exosomes from AML12 cells, loaded with CRISPR/Cas9-VP64 and sgRNA targeting HNF4α, inhibited hepatic stellate cell (HSC) activation by editing the HNF4α genome in hepatocytes. Targeting HSCs with nanocarriers is a promising anti-fibrotic strategy (Luo et al., 2021a). Cyclin-dependent protein kinase (CcnE1) promotes HSC proliferation (Nevzorova et al., 2012). In 2022, Wan et al. demonstrated that ribonucleoprotein complexes containing Cas9 and sgRNA targeting CcnE1 were packaged into exosomes derived from HSCs, which specifically localized to the liver (Wan et al., 2022). While exosomes offer advantages as delivery systems, tissue-specific targeting and potential side effects require further investigation. Additionally, the impact of genomic editing on normal hepatocytes remains unclear, and off-target effects of the CRISPR-Cas9 system pose challenges in gene therapy for liver fibrosis. Another group engineered RBP4-modified exosomes to deliver the CRISPR/dCas9 complex, inhibiting HSC activation and proliferation by targeting HNF4α/HGF1/FOXA2 genes, thereby converting myofibroblasts back to quiescent HSCs to combat liver fibrosis (Luo et al., 2022). Furthermore, exosomes have emerged as a promising vehicle for delivering antiviral agents. CRISPR technology has been successfully utilized to engineer exosomes for precise genomic editing, targeting viral replication in liver diseases such as hepatitis B and C (Ramanan et al., 2015). CRISPR-based designer nucleases specifical targeting HBV cccDNA reported in our previous publication (Zhang et al., 2023a). Additionally, exosomes loaded with epigenetic modifiers have shown potential in altering viral gene expression, providing a novel approach to antiviral therapy (Gheitasi et al., 2024). In conclusion, engineered exosomes have demonstrated significant therapeutic potential as delivery systems for LF treatment as shown in Table1. Growing emphasis is being placed on harnessing exosomes for targeted interventions to prevent or reverse LF progression. Future research should focus on optimizing exosome engineering for enhanced specificity and efficacy, ultimately paving the way for innovative therapeutic solutions in liver fibrosis management (Kalluri and LeBleu, 2020).

## 7 Nervous disorders and exosomes

Nervous disorder has a wide effect on human health in the world. Although surgical intervention and medical treatment can temporarily relieve suffering from this disease, those approaches cannot cure them completely. Thus, the effective cure for neurological diseases remains a big problem in the medical field (Silberberg et al., 2015). Therefore, it is an unmet need to explore effective and novel therapies for these diseases. Recently, exosomes have been identified to play an advantageous role in the development of nervous disorders like Parkinson’s disease, Alzheimer’s disease, and depression. Exosomes are the leading area of interest because they participate in not only promoting misfolded proteins that can cause nervous disorders, but also unfolding folded proteins in the brain (Kalluri and LeBleu, 2020). Moreover, not only can exosomes aid the unfolding of the folded protein, but they can also provide a biomarker for early diagnosis of nervous disorders as well because of their function in transferring lipids, proteins, and RNAs that serve as bio-information among tissues (Ye et al., 2020). Patients diagnosed with Parkinson’s disease have a higher level of α-synolig and a lower level of α-syntotal in their salivary exosomes than in healthy subjects (Rastogi et al., 2021). Exosomes’ capability of harboring small fingerprints of their host cell type allows for earlier and faster diagnosis (Soares Martins et al., 2021). Because of the exosome’s unique capacity to function as a carrier, they also hold the potential to be able to treat or alleviate nervous disorders. In studies performed on mice, it was found that exosomes that are packaged with miR-207 can efficiently and effectively alleviate symptoms of depression by directly targeting NF-kB signaling in astrocytes (Li et al., 2020b). Exosomes derived from neuroblastoma can mediate the clearance of brain Aβ by preventing the transporting of Aβ peptides to microglia in Alzheimer’s disease using a mouse mode *in vivo* (Guo et al., 2021). Excitingly, one group demonstrated that exosomes bidirectionally move across the blood-brain barrier (BBB). They confirmed the presence of proteins specifically released by astrocytes from the blood in rats within exosomes, which indicates exosomes can penetrate from the multiple layers of CNS to the peripheral circulation (Osaid et al., 2023).

**FIGURE 7:**
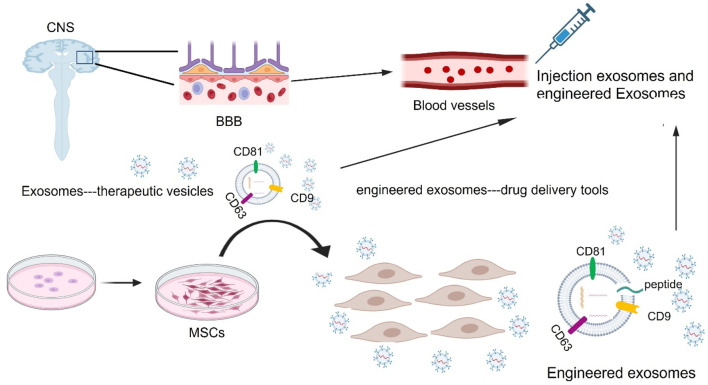
Exosomes as drug delivery vehicles and therapeutic targets for treating neurological disorders. During *in vivo* systems, exosomes can carry their own substances or therapeutic exogenous cargoes including DNA, RNAs, lipids, and proteins through the blood-brain barrier (BBB) into the damaged sites of the central nervous system (CNS), with low toxicity and immunogenicity. MSC: mesenchymal stem cells.

Over the years the efficiency of exosome targeting has improved in brain diseases tremendously. A study focused on exosomes engineered with cyclo peptide (RGDyK) demonstrated that the concentration of modified exosomes with high affinity to integrin αvβ3 in brain lesions is tenfold higher when compared to undamaged tissue on the adjacent side of the brain (Terstappen et al., 2021; Li et al., 2021). From a therapeutic point of view, mesenchymal stem cell-derived exosomes have been identified as a possible therapeutic treatment to reduce neuroinflammation and promote neurogenesis for Parkinson’s disease (Pinnell et al., 2021) as shown in Figure 7. In summary, exosomes apply for therapeutic roles mainly by delivering drugs as a tool or by their natural therapeutic characteristics of treatment in neurological disorders as shown in Table1. Despite all the advantages exosomes provide, exosome research still requires solving some limitations such as standardized guidelines for the isolation of exosomes (Babaei and Rezaie, 2021). Also, with the progression of clinical trials in the near future, it is hopeful to overcome many of the limitations placed on exosomes such as the understanding of how to control all the different repair pathways involved in therapeutic treatments (Li et al., 2018a).

## 8 Current and future translational capabilities of exosomes

Exosomes have emerged as promising candidates for therapeutic and diagnostic applications due to their natural ability to transport bioactive molecules, cross biological barriers, and mediate intercellular communication. The translational potential of exosomes has been increasingly recognized, leading to a surge in research efforts aimed at harnessing their capabilities for clinical applications. Currently, numerous clinical trials are investigating the potential of exosome-based therapies across various medical conditions, including immune disease, neurodegenerative disorders, cardiovascular diseases, and regenerative medicine. According to ClinicalTrials.gov, as of 2024, over 100 clinical trials are exploring exosome-based interventions. In immune diseases, Exosomes derived from mesenchymal stem cells are being tested for their ability to deliver chemotherapeutic agents or immune-modulating molecules to enhance cancer treatment. In neurological disorders, trials are assessing exosomes’ potential in treating conditions like Alzheimer’s disease, Parkinson’s disease, and stroke, focusing on their neuroprotective and regenerative properties (Fayazi et al., 2021). In regenerative medicine, several studies are evaluating exosome-based therapies for tissue repair in conditions such as osteoarthritis (Olumesi and Goldberg, 2023; Bai et al., 2024), myocardial infarction (Zheng et al., 2022), and skin wound healing (Rasti et al., 2024). Several organizations and biotech companies are driving innovation in exosome research and translation. Some of the leading institutions include: Codiak BioSciences is a pioneer in engineering exosomes for targeted drug delivery. Evox Therapeutics is focused on exosome-based drug delivery systems for genetic disorders. Aruna Bio is specializes in exosome therapies for neurological disorders. Exosome Diagnostics (a subsidiary of Bio-Techne) works on liquid biopsy applications for cancer diagnostics. Harvard Stem Cell Institute & Mayo Clinic is conducting extensive research on exosome-based regenerative medicine.

## 9 The future challenges and opportunities

Despite the significant progress of exosomes in treating various diseases, the transition of exosomes into clinical application has been hindered by several limitations, including low production yield, potential off-target effects, lack of standardized quality control, and limited clinical trials (Ye et al., 2020). The time-consuming process and high costs associated with exosome isolation and purification further impact their feasibility for large-scale therapeutic applications (Chen et al., 2021b). Additionally, challenges remain in enhancing exosomes packaging efficiency and targeting specificity to ensure effective therapeutic delivery. In additional, the heterogeneity of exosomes and variations in their cargo composition also pose challenges in ensuring reproducibility and consistency in therapeutic applications. Furthermore, the potential immunogenicity and unforeseen side effects of exosome-based treatments require thorough investigation before clinical implementation. Recent advancements have demonstrated promising solutions to overcome these challenges. Gene expression manipulation has shown potential in increasing exosome production, mitigating the limitation of low yield. Engineering parent cells, such as dendritic cells (DCs) (Lee and Friedman, 2011), has been explored to improve exosome targeting specificity. Furthermore, CRISPR/Cas9 technology has emerged as a tool to enhance exosome-based therapies, allowing precise genetic modifications to optimize therapeutic efficacy. Additionally, the development of scalable bioprocessing techniques, such as microfluidic-based exosome isolation and purification, is expected to improve production efficiency. Advances in bioengineering, including surface modifications and cargo loading techniques, are also enhancing exosome stability and delivery precision. Additionally, addressing public concerns about gene editing and ensuring the ethical application of technologies like CRISPR/Cas9 will be essential in advancing exosome-based treatments in liver dieases (Zhang et al., 2023a). In future, the increased collaboration between academia, industry, and regulatory agencies will be critical in accelerating clinical translation. With continued research and innovation, exosomes will have the more potential to revolutionize targeted drug delivery, regenerative medicine, and personalized therapeutic strategies.

## 10 Conclusion

Exosome-mediated delivery holds significant potential as a natural and efficient system for targeted drug and biomaterial transport. Their ability to carry customized lipids, DNA, RNA, and proteins makes them ideal candidates for targeted delivery, while also highlighting their key role in cell-cell communication. Despite current limitations, such as suboptimal targeting, low yield, and inefficient packaging, ongoing advancements in exosome production and engineering are addressing these challenges. Continued progress in exosome optimization and clinical validation will be essential for their successful transition from research to widespread therapeutic use. This article has explored the biogenesis, functions, and promising applications of exosomes, with the goal of providing clarity and inspiring further exploration of exosomes as a novel and innovative approach to combating human diseases and disorders.
